# Surgical outcomes of endoscopic dacryocystorhinostomy: analysis of age effect

**DOI:** 10.1038/s41598-019-56491-y

**Published:** 2019-12-27

**Authors:** Jae Yun Sung, Yeon Hee Lee, Kyoung Nam Kim, Tae Seen Kang, Sung Bok Lee

**Affiliations:** 1 Department of Ophthalmology, Chungnam National University College of Medicine, Chungnam National University Hospital, Daejeon, Republic of Korea; 2 Department of Ophthalmology, Kyeongsang National University Changwon Hospital, Changwon, Republic of Korea

**Keywords:** Lacrimal apparatus diseases, Eyelid diseases

## Abstract

There is limited evidence in literature determining age effect on outcomes of endoscopic dacryocystorhinostomy (EDCR) in adult patients with primary acquired nasolacrimal duct obstruction (NLDO). We aimed to analyze the outcomes of EDCR according to age in primary acquired NLDO. A retrospective study was performed on consecutive adult patients and patients were divided into two age groups; group 1 (aged to 61 years) and group 2 (aged 62 to 89 years) based on the average value. The minimum required follow-up period was 6 months. A total of 441 EDCRs performed in 342 patients were enrolled. The anatomical success rate was not significantly different between the two groups (91.8% and 88.2%, *P* = 0.209). However, the functional success rate was significantly lower in the group 2 (85.1% and 76.9%; *P* = 0.036). Functional failure was associated with old age and a history of diabetes mellitus (*P*  = 0.024 and *P*  = 0.008). In subgroup analysis of patients with anatomical success but functionally failed EDCR, group 2 had significantly more comorbid conditions such as eyelid laxity (*P* = 0.026). In conclusion, the comorbid conditions which increase with age may affect functional outcome, especially eyelid laxity, careful preoperative examination of the eyelid and conjunctiva should be emphasized to lacrimal surgeons before performing EDCR.

## Introduction

Endoscopic dacryocystorhinostomy (EDCR) is a currently well-established surgical treatment for patients with nasolacrimal duct obstruction (NLDO). In the past, external dacryocystorhinostomy (DCR) was considered as the gold standard treatment for NLDO. Over the past decade, EDCR has evolved as an alternative treatment option with significant advantages and high success rate. The advantages of EDCR over external DCR include a lack of cutaneous scarring, preservation of the pump function of the orbicularis oculi muscle, minimal risk of bleeding and rapid postoperative recovery time. With advances in nasal endoscopes and surgical instruments, EDCR has achieved high success rate comparable to that of external DCR. In recent studies, the overall success rates of EDCR are reported to range 82% to 98%^1–6^.

Various factors have been reported to affect surgical outcomes of EDCR in patients with NLDO, such as duration of intubation, pre-existing sinus or nasal abnormalities, previous trauma or nasal surgery and revision for failed DCR^6–10^. However, there is limited evidence in the literature determining age effect on the surgical outcome of EDCR in adult patients with primary acquired NLDO. Zenk *et al*.^9^ and Dolmetsch^10^ found that age had no significant effect on the success of EDCR, but various etiology of NLDO (congenital, primary and secondary NLDO) were included. In this study, we analyzed the anatomical and functional outcomes of EDCR by age in a large adult population with primary acquired NLDO. We also investigated the risk factors associated with functional failure.

## Results

A total of 441 EDCRs performed in 342 patients were enrolled in this study, including 220 cases in group 1 and 221 cases in group 2. The mean age of the whole study group at surgery was 61.2 years (range, 30 to 87). The female to male ratio was 5.1:1. Of the 342 patients, 243 underwent unilateral surgery and 99 underwent bilateral surgery. The mean duration of symptoms was 35.5 ± 37.3 months. Demographics of all patients are demonstrated in Table 1.

**Table 1:** Demographics of the 342 patients.

DCR procedures (n)	441
Age (years, mean ± SD)	61.2 ± 11.4 (30–87)
**Gender** (**n**, **%**)	
Female	369 (83.7)
Male	72 (16.3)
**Laterality** (**n**, **%**)	
Right	210 (47.6)
Left	231 (52.4)
Bilateral	99 (22.4)
**Medical history** (**n**, **%**)	
Hypertension	156 (35.4)
Diabetes mellitus	49 (11.1)

DCR = dacryocystorhinostomy; SD = standard deviation.

The mean age of the group 1 and group 2 was 51.9 years and 70.5 years. There were no significant differences between groups in terms of gender and laterality (*P* = 0.427 and *P* = 0.884). The number of patients with medical history of hypertension and diabetes mellitus increased with age (*P* < 0.001 and *P* = 0.002). (Table 2).

**Table 2:** Demographics of the 342 patients subdivided by age group.

	Group 1 (30–61 years)	Group 2 (62–89 years)	*P*-value
DCR procedures (n)	220	221	
Age (years, mean ± SD)	51.9 ± 7.1	70.5 ± 6.0	
Gender (n, %)			0.427^*^
Female	181 (82.3)	188 (85.1)	
Male	39 (17.7)	33 (14.9)	
Laterality (n, %)			0.884^*^
Right	104 (47.3)	106 (48.0)	
Left	116 (52.7)	115 (52.0)	
**Medical history (n, %)**			
Hypertension	51 (23.2)	105 (47.5)	<**0**.001*
Diabetes mellitus	14 (6.4)	35 (15.8)	**0**.002*

DCR = dacryocystorhinostomy; SD = standard deviation; Significant P values are expressed in bold characters.

^*^Chi-square test.

### Surgical outcomes

At 6 months after surgery, the overall anatomical and functional success rate was 90.0% (397/441) and 73.0% (322/441) respectively. The anatomical success rate was 91.8% (202/220) in group 1 and 88.2% (195/221) in group 2, with no significant difference (*P* = 0.209). The functional success rate was 85.1% (172/202) in group 1 and 76.9% (150/195) in group 2. The functional success rate was significantly lower in group 2 (*P* = 0.036).

EDCR with uncinectomy was performed in 145 cases (32.9%) and EDCR with middle turbinectomy was performed in 101 cases (22.9%). There was no significant difference in the number of DCR procedures performed with uncinectomy or middle turbinectomy between the 2 groups. At 4 months after surgery, ostium granulation was observed in 66 cases (15.0%), membranous obstruction in 19 cases (4.3%) and intranasal synechiae in 11 cases (2.5%). There was no significant difference in the occurrence rate of ostium granulation, membranous obstruction or intranasal synechiae between the 2 groups. No significant complications were noted, such as orbital fat prolapse, cerebrospinal fluid leak, infection or delayed severe epistaxis.

### Analysis of factors affecting functional outcomes

Of the 397 anatomically successful cases, 75 (18.9%) did not show functional success (functional failure). The influence of preoperative, intraoperative and postoperative factors on the functional outcome was evaluated. Age and history of diabetes mellitus were significantly associated with functional outcomes. The functional failure group showed older age and a higher prevalence of diabetes mellitus than the functional success group (*P* = 0.024 and *P* = 0.008). Other factors including gender, laterality, hypertension, uncinectomy, middle turbinectomy, ostium granulation, membranous obstruction and intranasal synechiae were not significantly associated with functional outcomes (*P* > 0.05) (Table 3).

**Table 3:** Comparison of demographics, intraoperative factors and postoperative factors between the functional success and functional failure in 397 anatomically successful cases.

	Functional success	Functional Failure	*P*-value
DCR procedures (n, %)	322 (81.1)	75 (18.9)	
Age (years, mean ± SD)	60.5 ± 11.4	63.9 ± 11.8	**0**.024^*^
Gender (n, %)			0.119^†^
Female	273 (84.8)	58 (77.3)	
Male	49 (15.2)	17 (22.7)	
Laterality (n, %)			0.429^†^
Right	158 (49.1)	33 (44.0)	
Left	164 (50.9)	42 (56.0)	
**Medical history (n, %)**			
Hypertension	111 (34.5)	33 (44.0)	0.122^†^
Diabetes mellitus	27 (8.4)	14 (18.7)	**0**.**008**^†^
**Intraoperative factors (n, %)**			
Uncinectomy	103 (32.0)	31 (41.3)	0.123^†^
Middle turbinectomy	74 (23.0)	20 (26.7)	0.499^†^
**Postoperative factors (n, %)**			
Ostium granulation	38 (11.8)	10 (13.3)	0.714^†^
Membranous obstruction^a^	1 (0.3)	2 (2.7)	0.093^‡^
Intranasal synechiae	9 (2.8)	0 (0.0)	0.218^‡^

DCR = dacryocystorhinostomy; SD = standard deviation; Significant P values are expressed in bold characters.

^*^Student’s t-test.

^†^Chi-square test.

^‡^Fisher’s exact test.

^a^Membranous obstruction was evaluated at 4 months after surgery (1 month after silicone tube removal). When membranous obstruction was observed, gentle probing was performed to perforate the membrane, and the surgical outcome was assessed at 6 months after surgery.

When the 397 anatomically successful cases were analyzed by age group, 14.9% (30/202) of the cases in group 1 and 23.1% (45/195) in group 2 showed functional failure (*P* = 0.036). To analyze the cause of anatomical success but functionally failed DCR cases, comorbid conditions such as eyelid laxity, conjunctivochalasis, and everted punctum were evaluated in 75 cases. The two most common conditions were eyelid laxity and conjunctivochalasis. Eyelid laxity was combined in 23.3% (7/30) of cases in group 1 and 48.9% (22/45) in group 2 (*P* = 0.026). Conjunctivochalasis was combined in 30.0% (9/30) of cases in group 1 and 37.8% (17/45) in group 2 (*P* = 0.488). Everted punctum was observed in 3.3% (1/30) of cases in group 1 and 6.7% (3/45) of cases in group 2 (*P* = 0.646). The prevalence of comorbid conditions which can affect the functional outcome increased with age; especially, proportion of patients with eyelid laxity was significantly higher in group 2 (Table 4).

**Table 4:** Comorbid conditions in 75 cases of functional failure by age group.

	Group 1 (30–61 years)	Group 2 (62–89 years)	*P* value
Functional failure (n, %)	30/202 (14.9)	45/195 (23.1)	**0**.036^*^
**Comorbid conditions** (**n**, **%**)			
Eyelid laxity	7 (23.3)	22 (48.9)	**0**.026^*^
Conjunctivochalasis	9 (30.0)	17 (37.8)	0.488^*^
Everted punctum	1 (3.3)	3 (6.7)	0.646^†^

DCR = dacryocystorhinostomy; Significant P values are expressed in bold characters.

^*^Chi-square test.

^†^Fisher’s exact test.

## Discussion

Olver^11^ suggested that there are three criteria that lacrimal surgeons should agree. First, assess the outcome at a minimum of 6 months after surgery, and at least 3 months after tube removal. Second, assess subjective success based on the patient’s symptoms. Third, assess objective success based on the patency on syringing and presence of functioning rhinostomy. We used above three criteria to evaluate the surgical outcomes. In this study, the overall anatomical success rate was 90.0% and the overall functional success rate was 73.0% at 6 months after surgery. Consistent with previous studies^2,12,13^, the functional success rate was lower than anatomical success rate. Functional success rate was relatively lower than previous studies, which reported success rates ranging from 81% to 96% after EDCR^2,4,9,14,15^. However, directly comparing the success rates of lacrimal surgery between studies is limited because different studies use different criteria of success, varying follow-up period and patient selection. We defined functional success as complete resolution of epiphora and did not include patients with partial improvement in the success group because these patients were still disturbed by tearing. The strict criteria of success may explain our relatively low functional success rate.

In this study, a large population of NLDO cases with a single etiology was included to clarify the effect of age on the outcome of standardized EDCR. Patients were divided into two age groups and surgical outcomes were evaluated. It is interesting to note that the anatomical success rate was comparable, while the functional success rate was significantly different between the two groups after EDCR. The disparity between anatomical success and functional success was greater in older patients. Attempts have been made to determine the prognostic factors for EDCR in patients with NLDO, and various factors have been reported such as duration of symptoms, duration of intubation, pre-existing sinus or nasal abnormalities, previous trauma or nasal surgery and revision for failed DCR^7,8,15^. However, little is known about the effect of age on the surgical outcome of EDCR in adult patients with primary acquired NLDO. Zenk *et al*.^9^ and Dolmetsch^10^ found that age had no significant association with the success of EDCR; however, cases varing in NLDO etiology (congenital, primary and secondary NLDO) were included in their study. Mak *et al*.^3^ evaluated prognostic factors of 83 DCRs performed for primary acquired NLDO; in contrast to our study, they reported that younger patients had a higher rate of failure and postulated the reason may be related to the higher degree of fibrosis in younger patients. However, their study was based on a relatively small sample size, especially failed group included only 5 cases. Tooley *et al*.^16^ compared the resolution of symptom rate between an elderly group (≥80 years) and a younger group (40–79 years) after 115 external DCRs. At the final follow-up, the symptom resolution rate was significantly lower in elderly patients versus younger patients (64% vs 86%), in agreement with our study. They explained that the older population may have more multiple conditions such as eyelid laxity and dry eye with reflex lacrimation which can lead to persistent symptoms. A high proportion of older patients may represent another possible reason for the relatively low functional success rate in our study.

The purpose of DCR is resolution of epiphora, however, some patients experience persistent symptoms despite anatomically successful DCR. The functional failure, persistent epiphora with patent ostium on syringing, has been reported to range from 1.7% to 4.7% after primary EDCR and from 5% to 12% after revision EDCR^14,17–19^. In a retrospective review of 61 failed EDCR procedures, Baek *et al*.^20^ reported functional failure in 15% of patients, and it was the third most common cause of EDCR failure. In this study, functional failure was found in 18.9% of patients after primary EDCR, which was a higher rate than in previous reports. We compared baseline demographics, intraoperative and postoperative factors in cases of functional failure to identify potential risk factors. Age at surgery and history of diabetes mellitus were significantly associated with functional failure. The high prevalence of diabetes mellitus in functional failure group may be a reflection of higher prevalence of diabetes mellitus in older patients. Therefore, age is considered to be a major factor affecting functional failure. The association between old age at surgery and functional failure may be related to the age-related changes in the globe and eyelid in older patients. We evaluated eyelid laxity, conjunctivochalasis and everted punctum in 75 functional failure cases after EDCR. The two most common comorbidities were eyelid laxity and conjunctivochalasis, and the older patient group had significantly more comorbid conditions than younger group. This result is in agreement with an earlier study by Tooley *et al*.^16^ that compared the resolution of symptom rate after external DCR between an elderly group and a younger group. Elderly patients were shown to be likely to experience less symptoms relief after external DCR and had a higher rate of comorbid eyelid conditions. Therefore, when planning DCR for the treatment of NLDO, one may consider that the cause of epiphora may be multifactorial and NLDO may be accompanied by functional epiphora especially in older patients. Careful examination of eyelid and punctum using the eyelid distraction test and slit-lamp biomicroscopy would be needed preoperatively.

There have been few reports about the treatment of functional failure after EDCR in NLDO patients. Shams *et al*.^21^ conducted a multicenter retrospective study to investigate the management and outcomes of 65 cases of functional failure after external DCR or EDCR, 60% of patients had silicone tube reintubation with a 54% success rate, and 34% of patients had an eyelid-tightening procedure with a 50% success rate. Although patients with significant eyelid laxity were excluded from the study, lower eyelid tightening procedure successfully treated half of the functional epiphora patients. In our study, all patients underwent EDCR, but several studies have reported the results of concomitant eyelid or conjunctival surgery. Lee *et al*.^22^ evaluated the efficacy of simultaneous EDCR and lateral tarsal strip procedure for the treatment of 17 NLDO patients (29 eyes) with lower eyelid laxity. Anatomical and functional success was achieved in 89.5% and 86.2% of patients. Although the study was not a comparative design, the authors concluded that lateral tarsal strip applied simultaneously with DCR could be an effective tool to manage NLDO with horizontal eyelid laxity. Kim *et al*.^23^ compared the surgical outcomes of silicone tube intubation alone versus in combination with conjunctival resection in patients with NLD stenosis accompanied by conjunctivochalasis, and reported that the success rate, tear meniscus height, and patient’s satisfaction were all superior in the combined surgery group. To improve surgical outcomes after EDCR in older patients, simultaneous eyelid tightening procedure and/or conjunctival resection with EDCR may be helpful for the treatment of epiphora resulting from eyelid laxity, conjunctivochalasis and NLDO.

The strengths of this study included the large study population with single etiology of epiphora and a uniform surgical procedure performed by a single experienced surgeon. The limitations included the retrospective design. Because of this, it was not possible to directly identify the effect of eyelid and conjunctival problems on the surgical outcomes after EDCR. Further prospective studies will be needed to determine whether performing correction of lid laxity or conjunctivochalasis with EDCR can significantly increase the functional success rate.

In conclusion, our results show that EDCR is an effective and safe procedure without significant complications. Older age was an important risk factor for poor surgical outcome after EDCR in adult patients with primary acquired NLDO. The older patients tended to experience less symptom relief and more functional failure after EDCR than the younger patients, possibly due to age-related changes in the globe and eyelid. Therefore, even without significant lid laxity or conjunctivochalasis, careful inspection of the eyelid and conjunctiva should be emphasized to lacrimal surgeons before performing EDCR.

## Methods

A retrospective review of the medical records was performed on consecutive adult patients who underwent EDCR procedures for primary acquired NLDO between September 2012 and August 2018 at Chungnam National University Hospital. Indication for surgery was persistent epiphora and the diagnosis of NLDO was based on the result of lacrimal irrigation confirmed by dacryocystographic findings. The minimum required follow-up period after surgery was 6 months. Patients with (1) punctal occlusion or canalicular obstruction, (2) lacrimal hypersecretion due to ocular surface disease, (3) significant lower lid laxity (lateral distraction test >5 mm), conjunctivochalasis (blockage of the inferior punctum on primary gaze), or punctal ectropion (visible punctum on primary gaze), (4) previous history of lacrimal surgery were excluded. Patients were divided into two age groups; group 1 (aged 30 to 61 years) and group 2 (aged 62 to 89 years) based on the average value. The study protocol was approved by the Institutional Review Board of Chungnam National University Hospital. Informed consent was obtained from all patients. The study adhered to the tenets of the Declaration of Helsinki. The requirement for obtaining informed patient consent was waived due to the retrospective nature of the study.

### Surgical technique

All of the patients were operated under general anesthesia by a single experienced surgeon (S.B.L). Nasal mucosa was decongested by packing with 1:10,000 epinephrine-soaked gauze. The upper punctum was dilated and a 23-gauge vitrectomy illuminator was inserted through upper canaliculus into the lacrimal sac. After identifying the location of lacrimal sac in the nasal cavity by transillumination, the nasal mucosa of the transilluminated area was infiltrated with 1:100,000 epinephrine plus 2% lidocaine mixed solution. If necessary, procedures including middle turbinectomy and uncinectomy were performed to enlarge the nasal cavity. The incision in the nasal mucosa was made by Colorado monopolar cautery and mucosa was removed using elevator and ethmoid forceps. The thick frontal process of maxillary bone and thin lacrimal bone were removed by Smith-Kerrison rongeur. The illuminator was positioned horizontally tenting the medial wall of the lacrimal sac to ensure the sac was fully exposure. The lacrimal sac was opened vertically using a crescent knife and removed using ethmoid forceps. After confirming the patency of lacrimal passage by saline irrigation, bicanalicular silicone tube was inserted and free ends were tied together. At the end of the surgery, anastomosis site was packed with biodegradable synthetic polyurethane foam (Nasopore^®^).

All patients were prescribed eye drops (topical antibiotics and topical steroids) and nasal spray. The follow-up examination was scheduled at 1 and 3 weeks, 2, 3, 4 and 6 months after surgery. At each visit, lacrimal irrigation was performed and the ostium was evaluated by endonasal endoscopy. The silicone tube was removed at 3 months after surgery.

### Main outcome measures

Primary outcome measures included both the anatomical (objective) success rate and functional (subjective) success rate at 6 months after surgery. Anatomical success was defined as patent ostium, confirmed by lacrimal irrigation without regurgitation. Functional success was defined as the complete resolution of epiphora in anatomically successful cases. Functional failure was defined as persistent epiphora with an anatomically patent ostium.

Factors associated with surgical outcomes and significant complications were evaluated. The occurrence of ostium granulation, membranous obstruction and intranasal synechiae was evaluated at 4 months after surgery. Conjunctivochalasis was defined when the redundant loose conjunctiva mechanically blocked the inferior punctum in downgaze. Lower eyelid laxity was evaluated using lateral distraction test. The lateral distraction test was performed by pulling the medial part of the eyelid laterally along a horizontal line until it could no longer be pulled with sustained traction. Even if there was no significant lower lid laxity (lateral distraction test >5 mm), eyelid laxity was defined when the distracted punctum was pulled laterally 3 mm or more^24^. Even if there was no significant punctal ectropion (grossly visible punctum on primary gaze), everted punctum was defined when the lower punctum was not apposition to the globe and was visible on slit lamp examination^25^.

### Statistical analysis

Statistical analysis was performed using SPSS for Window version 22.0 (SPSS Inc, Chicago, IL, USA). The student’s t-test, chi-square test, Fisher’s exact test, and linear-by-linear association were used to analyze the patients’ characteristics and surgical outcomes. Logistic regression was performed to identify factors affecting surgical outcomes including age, gender, laterality, medical history, uncinectomy, middle turbinectomy, ostium granulation, membranous obstruction, and intranasal synechiae. P-values of <0.05 were considered to be statistically significant.

## Data Availability

Data supporting the findings of the current study are available from the corresponding author on reasonable request.
